# Frontal assessment battery and frontal atrophy in amyotrophic lateral sclerosis

**DOI:** 10.1002/brb3.707

**Published:** 2017-04-12

**Authors:** Tatsuhiro Terada, Jun Miyata, Tomokazu Obi, Manabu Kubota, Miho Yoshizumi, Kinya Yamazaki, Kouichi Mizoguchi, Toshiya Murai

**Affiliations:** ^1^ Department of NeurologyShizuoka Institute of Epilepsy and Neurological DisordersAoi‐kuShizuokaJapan; ^2^ Department of Biofunctional ImagingMedical Photonics Research CentreHamamatsu University School of MedicineHigashi‐kuHamamatsuJapan; ^3^ Department of PsychiatryGraduate School of MedicineKyoto UniversitySakyo‐kuKyotoJapan; ^4^ Brain Disorder Translational Research TeamDepartment of Functional Brain Imaging ResearchNational Institute of Radiological SciencesNational Institutes for Quantum and Radiological Science and TechnologyInage‐kuChibaJapan

**Keywords:** amyotrophic lateral sclerosis, frontal assessment battery, frontal cognitive impairment, motor impairment, respiratory failure, voxel‐based morphometry

## Abstract

**Objectives:**

To determine the potential utility of the frontal assessment battery (FAB) in assessing cognitive impairments in amyotrophic lateral sclerosis (ALS), we investigated the association between the FAB score and regional gray matter volume, and ascertained whether the regional brain alterations related to cognitive impairments occur in relatively mild stage of ALS.

**Materials and Methods:**

Twenty‐four ALS patients with a Mini‐Mental State Examination score of >23, a normal score on the Self‐Rating Depression Scale, little or no disturbance in speech and handling utensils on the ALS Functional Rating Scale (ALSFRS), and normal measures on respiratory tests (respiratory function test and arterial blood gas analysis), and two age‐matched normal control groups (one for FAB assessment and the other for brain morphometry) underwent FAB testing and structural magnetic resonance imaging. We applied voxel‐based morphometry to investigate the relationship between the FAB score and regional brain alteration, and assessed the relationship between the altered regional brain volume and ALSFRS or respiratory tests.

**Results:**

Frontal assessment battery scores were significantly lower in ALS patients than in normal controls. Volume reduction in the right orbitofrontal gyrus in ALS was correlated with a lower FAB score. There was no correlation between the right orbitofrontal gyrus volume and ALSFRS or respiratory tests.

**Conclusions:**

The results suggest that the FAB is an adequate tool for detecting cognitive impairments related to frontal lobe pathology in the relatively mild stage of ALS, independent of physical dysfunctions.

## 1 Introduction

Amyotrophic lateral sclerosis (ALS) is a neurodegenerative disease characterized by the progressive loss of upper and lower motor neurons leading to limb paralysis, dysphagia, dysarthria, and respiratory failure. Although ALS was once believed to affect only the motor system, ALS patients with frontal cognitive impairments similar to frontotemporal dementia (FTD) have been reported (Goldstein & Abrahams, 2013; Lomen‐Hoerth et al., 2003; Murphy, Henry, & Lomen‐Hoerth, 2007). Recent studies have shown pathological evidence of a strong association between ALS and FTD (Chang et al., 2005; Mezzapesa et al., 2007).

Previous authors have suggested that cognitive deficits might appear in ALS patients in varying degrees. A recent study proposed the following classification for frontotemporal syndromes in ALS: ALS‐FTD, which meets the Neary criteria for FTD (Neary et al., 1998); ALS with behavioral impairment (ALSbi), in which behavioral features are associated with frontotemporal dysfunction; and ALS with cognitive impairment (ALSci), in which impairment is seen in scores on neuropsychological tests sensitive to executive dysfunction (Goldstein & Abrahams, 2013; Murphy et al., 2007). The cognitive impairments in ALS are mainly associated with executive dysfunction. Thus far, dozens of studies using several neuropsychological tests have demonstrated the presence of frontal cognitive impairments in a substantial proportion of ALS patients without dementia (Goldstein & Abrahams, 2013; Lomen‐Hoerth et al., 2003). However, established tests of frontal cognitive function such as the Wisconsin Card Sorting Test or the Word Fluency Test are relatively time‐consuming, difficult, and not feasible for the elderly or for ALS patients with severe physical impairments (Goldstein & Abrahams, 2013). Therefore, cognitive impairments as assessed by neuropsychological tests of frontal function in ALS patients may reflect task difficulty due to motor impairment (Goldstein & Abrahams, 2013). Furthermore, because these tests were not designed to detect heterogeneous frontal cognitive involvement, no consensus has been reached on how to assess frontal cognitive impairment when distinguishing ALSci from patients traditionally characterized as ALS without dementia.

The frontal assessment battery (FAB) is a simple and short battery of neuropsychological tests for bedside screening of global executive dysfunction; it includes six subtests that explore different cognitive functions related to the frontal lobe (Dubois, Slachevsky, Litvan, & Pillon, 2000). To date, the FAB has been used to evaluate frontal lobe dysfunction in patients with ALS and other neurodegenerative disorders (Ahn et al., 2011; Dubois et al., 2000; Floris et al., 2012; Kume et al., 2011; Matsui et al., 2006; Nakaaki et al., 2007; Osborne, Sekhon, Johnston, & Kalra, 2014; Raaphorst et al., 2013), and the results suggest the presence of frontal cognitive impairments in ALS patients. However, an understanding of the underlying neuroanatomical changes is lacking despite the basic assumption that the regional volumes of particular brain areas should correlate with their functions (Ueda et al., 2010).

The aim of this study was to investigate the relationship between frontal cognitive dysfunctions detected by the FAB and brain volumes in ALS patients without dementia or depression, and to determine whether the anatomical changes associated with frontal cognitive dysfunctions are influenced by physical and emotional changes related to ALS.

## 2 Materials and methods

### 2.1 Subjects

Twenty‐four subjects with sporadic ALS (13 men and 11 women: mean age ± *SD*, 66.4 ± 10.6 years; mean disease duration 2.2 ± 1.7 years; all right‐handed) were recruited from the Shizuoka Institute of Epilepsy and Neurological Disorders (SIEND). All subjects met the criteria for the diagnosis of probable ALS, probable ALS laboratory‐supported, or definite ALS based on the El Escorial criteria (Beghi et al., 2002). The exclusion criteria were as follows: (1) subjects whose Mini‐Mental State Examination (MMSE) scores were <24, indicating obvious dementia (Tombaugh & McIntyre, 1992); (2) subjects whose Self‐rating Depression Scale (SDS) score were more than 60, reflecting obvious depression (Holmes, Fouty, Wurtz, & Burdick, 1988); (3) the presence of cerebrovascular disease, hydrocephalus, brain tumor, or traumatic brain injury; or (4) subjects with severe physical impairments or respiratory dysfunction, as described below in detail.

We recruited two groups of age‐matched healthy controls. All the healthy controls were right‐handed. The control group for FAB evaluation consisted of 70 healthy subjects (35 men and 35 women: mean age ± *SD*, 69.4 ± 5.8 years). The healthy control group for voxel‐based morphometry (VBM) analysis included 17 subjects (10 men and 7 women: mean age ± *SD*, 69.4 ± 5.8 years). None of the controls had any neurological problems, history of head injury, psychiatric disease, serious medical illness, or major surgery. Their Clinical Dementia Rating (CDR) score of zero indicated a lack of dementia (Hughes, Berg, Danziger, Coben, & Martin, 1982). No significant differences in age, gender, and behavior assessment (by CDR) were found between the two groups of healthy controls.

This study was reviewed and approved by the ethics committee of SIEND. All subjects gave informed consent to participate in the study.

### 2.2 ALS‐related physical assessment

ALS‐related physical impairment was evaluated using the revised ALS Functional Rating Scale (ALSFRS) (Cedarbaum et al., 1999). All patients met the following inclusion criteria: (1) the speech subscore was 4 (normal speech process) or 3 (detectable speech disturbance but intelligible without repeating); (2) the cutting food and handling utensils subscore was 4 (normal praxis in eating) or 3 (somewhat slow and clumsy, but no help needed); (3) the dyspnea subscore was 4 (no dyspnea); (4) the orthopnea subscore was 4 (no orthopnea); and (5) the respiratory insufficiency subscore was 4 (no use of biphasic positive airway pressure or invasive mechanical ventilation by intubation or tracheostomy).

Upon enrollment, four of the 24 ALS patients were bulbar onset, and the remaining 20 patients were patients with spinal onset. All patients met the following inclusion criteria using ALSFRS: (6) the salivian subscore was 4 (normal) or 3 (slight excess of saliva in mouth); (7) swallowing subscore was 4 (normal eating habit) or 3 (early eating problems, occasional choking).

The ventilatory status of each patient was evaluated by a standard respiratory function test (forced vital capacity [FVC] and FVC%, adjusted for each subject's gender, age, and height) and arterial blood gas analytes (pCO_2_, pO_2_, HCO_3_, and Base Excess). The exclusion criteria were as follows: (1) subjects with reduced vital capacity (FVC% < 80); (2) subjects with hypoxemia (pO_2_ < 70 mmHg); or hypercapnia (pCO_2_ > 45 mmHg).

### 2.3 Frontal cognitive evaluation

We used the Japanese version of the FAB, which has been validated as a reliable screening test (Kugo et al., 2007; Nakaaki et al., 2007). The FAB consists of six subtests: similarity, lexical fluency, motor series, conflicting instructions, go–no go, and prehension behavior. The score for each subtest ranges from 0 to 3. These scores are added to calculate the total FAB score; hence, the maximum total score is 18. Higher scores reflect better performance (Dubois et al., 2000). Because age has significant effects on the FAB score, the age of the subjects should be taken into consideration when evaluating the results (Appollonio et al., 2005). Therefore, we administered the FAB to both ALS patients and age‐matched healthy controls.

In addition, we investigated the frontal lobe‐mediated behavioral dysfunction in ALS patients using observer‐rating behavioral assessment, the Japanese version of the family‐rating Frontal Systems Behavioral Scale (FrSBe) (Grace, Stout, & Malloy, 1999; Yoshizumi, Ueda, Ohigashi, & Murai, 2007). The patient's current behavior was scored, and raw scores of FrSBe can be transformed into standard T scores adjusted for age and gender. Total score and three subscale for apathy, disinhibition, and executive dysfunction were calculated. The mean T score corresponded to 50 and the standard deviation was set to 10, and mean ± 1*SD* of the normative value was termed the normal range in this study. High scores indicate higher level of behavioral impairments (Grace et al., 1999; Yoshizumi et al., 2007).

### 2.4 MRI acquisition

All participants underwent MRI scans on a 1.5‐T scanner (General Electric Healthcare, UK) equipped with a standard quadrature head coil. Three‐dimensional T1‐weighted images were acquired and T2‐FLAIR images were reviewed to exclude potential abnormalities in all participants as described above. The scanning parameters for T1‐weighted images were as follows: TR = 17.0 ms; TE = 3.0 ms; flip angle 20°; acquisition matrix = 256 × 192; FOV = 240 mm; 124 contiguous sagittal sections, each with a thickness of 1.3 mm; and a resolution of 0.9375 mm × 1.25 mm × 1.3 mm.

### 2.5 Statistical analysis

The behavioral data were analyzed using the Statistical Package for Social Sciences version 17 (SPSS Inc, Chicago, IL, USA). Comparison of the FAB scores between the ALS patients and the control group were evaluated with *t*‐tests. Correlations between FAB scores and ALS‐related clinical factors (disease duration, ALSFRS, and ventilatory status) were investigated using Pearson correlation coefficients. The criterion for statistical significance was set at *p *< .05. All data are presented in the form of mean ± 1 standard deviation (*SD*).

### 2.6 Imaging data processing

We applied VBM to investigate regional changes in brain volume, using Statistical Parametric Mapping 8 (SPM8; Wellcome Department of Imaging Neurosciences, London, UK; http://fil.ion.ucl.ac.uk/spm) software. Specifically, we used The Diffeomorphic Anatomical Registration Through Exponentiated Lie algebra (DARTEL) toolbox running on Matlab 7.5.0 (The MathWorks, Natick, MA, USA). This procedure has been described elsewhere (Kubota et al., 2011; Terada et al., 2016). Three‐dimensional T1‐weighted images were segmented into gray matter, white matter, cerebrospinal fluid, skull, soft tissue, and air/background, using the “New Segment” algorithm in SPM8. The DARTEL approach was used to nonlinearly deform gray and white matter segments and create a series of custom templates from the images of all participants. Nonmodulated gray and white matter segments were normalized to Montreal Neurological Institute (MNI) 152 space and smoothed by a 12‐mm full‐width‐at‐half‐maximum Gaussian kernel, which has often been used in VBM studies (Miyata et al., 2009; Ueda et al., 2010).

### 2.7 Regional gray matter reduction in ALS patients relative to controls

**Figure 1:**
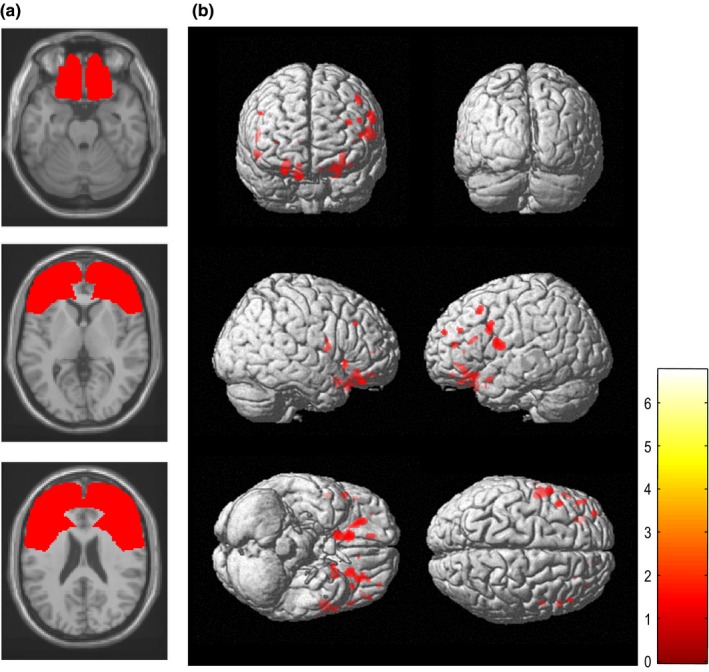
(a) Illustration of the frontal lobe mask (shown in red) created using the WFU pick atlas, superimposed on the brain MRI. (b) Gray matter volume reductions in patients with amyotrophic lateral sclerosis relative to controls (statistical threshold set at *p *<* *.001, uncorrected) using the frontal lobe mask. The results are displayed as three‐dimensionally rendered brain surface images. Patients showed significant gray matter reductions (shown in red) in the bilateral frontal cortex

Two‐sample *t*‐tests were used to identify brain areas where the ALS group showed a reduction in gray matter relative to the control group in SPM8. Age, gender, and total gray matter volume were treated as covariates of no interest. The voxel‐level height threshold was set at *p *<* *.001 (uncorrected) in this exploratory analysis. Because the regions of focus were known a priori as areas with ALS pathology according to the previous pathologic and neuroimaging studies (Chang et al., 2005; Mezzapesa et al., 2007), we explored only within frontal lobe regions. Therefore, a frontal lobe mask was created anatomically using WFU pick atlas version 2.52 toolbox (http://fmri.wfubmc.edu/software/PickAtlas) which is available in SPM8 (Figure 1a), and two‐sample *t*‐tests were undertaken using the frontal lobe mask. The result of this group comparison within frontal lobe was used as the final inclusion mask in the following correlation analysis (Figure 1b). In addition to analyzing gray matter reduction, we analyzed regions in which gray matter increased in the ALS group compared with the control group.

### 2.8 Correlation analysis

Correlations were analyzed in SPM8 to identify the brain region that positively correlated with the total FAB score of ALS patients. The above mentioned mask was used to include only those voxels with significant group differences in the frontal lobe (Figure 1b). Age, gender, and total gray matter volume were entered in the design matrix as covariates of no interest. Controls were not subjected to correlation analysis because their FAB scores were not expected to show sufficient variation. The threshold for statistical significance was set at *p *<* *.05 (family‐wise error [FWE] rate correction at the cluster level). MNI coordinates were transformed into Talairach coordinates (Talairach & Tournoux, 1988), using the mni2tal.m Matlab script (http://imaging.mrc-cbu.cam.ac.uk/imaging/MniTalairach).

In addition, we further examined the correlation between regional brain volumes and FAB subset scores. Each ALS patient's regional gray matter volume (eigenvariate) for a cluster of significant correlations was extracted using the VOI (volume of interest) function in SPM8. We analyzed the correlation between each eigenvariate and each FAB subtest score using Pearson correlation coefficients (six‐subtest of FAB × one cluster of significant correlations) in SPSS. Furthermore, correlations were analyzed using Pearson correlation coefficients between each eigenvariate and ALS‐related clinical factors (ALSFRS, respiratory function, arterial blood gas analytes, MMSE, and SDS). Since these analyses were exploratory, no Bonferroni corrections were used. As a rule of thumb, a sample size‐to‐number of independent variable ratio of 10 was required for multiple regression analysis. Therefore, multiple regression analysis was not used because the sample size was relatively small.

## 3 Results

Demographic and ALS‐related clinical factors for the ALS patient and control groups are presented in Table 1.

**Table 1:** Demographic and clinical characteristics of the amyotrophic lateral sclerosis (ALS) patient group and control groups

	ALS patient group (*n *= 24)	Control group for FAB (*n *= 70)	Control group for VBM (*n *= 17)	Normal range	*p* value
Age (years)	66.4 ± 10.6 (44–87)	62.1 ± 9.6	69.4 ± 5.8		n.s.
Men/women (number)	13/11	35/35	10/7		n.s.
Disease duration (years)	2.2 ± 1.7 (1–3)				
ALS functional rating scale (/48)	40.5 ± 2.1 (38–44)				
Mini‐Mental State Examination (/30)	27.2 ± 2.2 (24–30)			>23	
Self‐rating Depression Scale (/100)	46.2 ± 9.6 (24–59)			<60	
Respiratory function test					
Forced vital capacity (L)	2.94 ± 0.80 (1.70–4.86)				
% forced vital capacity (%)	99.2 ± 14.5 (82.7– 129.8)			>80	
Vital capacity (L)	2.97 ± 0.79 (1.98–4.89)				
% vital capacity (%)	100.6 ± 13.2 (82.9– 129.1)			>80	
Arterial blood gas analytes					
pCO2 (Torr)	41.4 ± 2.1 (38.1–44.8)			35–45	
pO2 (Torr)	89.1 ± 10.3 (81.1–107.6)			70–100	
HCO3 (mEq/L)	27.3 ± 1.5 (25.0–30.4)			22–26	
Base excess (mEq/L)	3.2 ± 1.7 (0.6–6.5)			−2 to +2	
FrSBe					
Total score	53.2 ± 10.6			40–60	
Apathy subscore	61.7 ± 11.2			40–60	
Disinhibition subscore	49.0 ± 8.3			40–60	
Executive dysfunction subscore	48.8 ± 11.0			40–60	
FAB (/18)	13.3 ± 1.8	15.0 ± 1.3			<.001^a^
Subtest score (/3)					
1. Similarities	1.2 ± 0.8	1.5 ± 0.7			.032^a^
2. Lexical fluency	2.0 ± 0.7	2.7 ± 0.5			<.001^a^
3. Motor series	2.8 ± 0.7	2.9 ± 0.3			.691
4. Conflicting instructions	2.6 ± 1.0	2.8 ± 0.5			.759
5. Go–No‐Go	1.6 ± 1.0	2.2 ± 0.8			.014^a^
6. Prehension behavior	3.0 ± 0.2	2.9 ± 0.3			.776

VBM, voxel‐based morphometry; ALS, amyotrophic lateral sclerosis; FrSBe, Frontal Systems Behavior Scale; FAB, Frontal Assessment Battery.

Data are presented as mean ± *SD* (range).

a *p *<* *.05.

### 3.1 Frontal cognitive ratings and ALS‐related physical variables

The total FAB score in patients with ALS was significantly lower than in normal controls. In individual subtests of the FAB, the similarity, lexical fluency, and go–no‐go scores were significantly lower in ALS patients than in normal controls (Table 1). The apathy subscale of the FrSBe was above the normal range, indicating apparent behavioral dysfunction (Table 1).

There were no significant correlations between the total FAB score and ALS‐related clinical factors, including disease duration, ALSFRS, and ventilatory status (forced vital capacity, FVC%, pCO2, pO2, HCO3, and Base Excess). Furthermore, no significant correlations were found between ALS‐related clinical factors and the similarity, lexical fluency, or go–no‐go score.

### 3.2 Regional gray matter reduction in ALS patients relative to controls

Patients with ALS showed significant gray matter reduction relative to controls, predominantly in the bilateral frontal cortex (*p *<* *.001, uncorrected) (Figure 1b and Table 2a). There were no regions in which gray matter significantly increased in ALS relative to controls.

**Table 2:** (a) Regions of gray matter volume reduction in patients with amyotrophic lateral sclerosis relative to controls. (b) Region of gray matter positively correlated with total Frontal Assessment Battery score in patients with amyotrophic lateral sclerosis

Anatomical region	BA	Talairach coordinates			Cluster size	Voxel T‐value	Z score	Cluster *p* value			Uncorrected voxel *p* value
		x	y	z				FWE	FDR	Uncorrected	
(a)											
Left precentral gyrus	6	−52.5	3.6	13.6	226	6.20	5.08	.222	.125	.013	<.001
Right rectal gyrus	11	11.9	22.1	−23.8	511	5.40	4.60	.011	.011	.001	<.001
Left middle frontal gyrus	11	−22.8	24.4	−16.4	237	4.71	4.13	.196	.125	.012	<.001
(b)											
Right orbitofrontal gyrus	11	23.8	36.8	−22.0	4	4.70	3.78	.029	.565	.565	<.001

BA, Brodmann's area; FWE, family‐wise error; FDR, false discovery rate.

### 3.3 Correlation analysis

**Figure 2:**
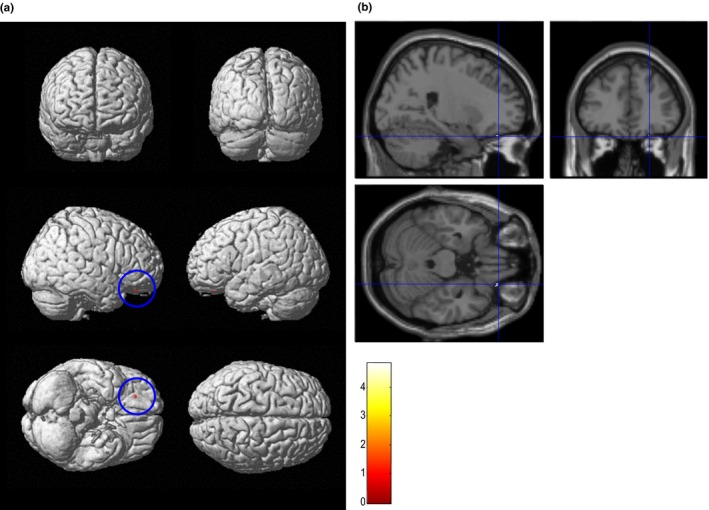
Region of gray matter volume positively correlated with total Frontal Assessment Battery (FAB) score in patients with amyotrophic lateral sclerosis (statistical threshold set at *p *<* *.05, family‐wise error, corrected). The results are displayed as three‐dimensionally rendered brain surface images (a) and T1 images (b). The total FAB score was significantly correlated with the right orbitofrontal gyrus volume (shown in red within the blue circle in a or at the intersection of the blue line in b)

The right orbitofrontal gyrus was identified as the region in which the total FAB score was positively correlated with gray matter volume in the ALS patient group (*p *<* *.05, FWE corrected; Figure 2 and Table 2b). Of the individual FAB subtest scores, the go–no‐go score (*r *= .452, *p *=* *.027), and motor series score (*r *= .441, *p *=* *.031) showed a significant positive correlation with the eigenvariate of the right orbitofrontal gyrus cluster. No significant correlations were found between the eigenvariate of the right orbitofrontal gyrus cluster and ALS‐related clinical factors, including disease duration, ALSFRS score, ventilatory status, MMSE score, or SDS score.

## 4 Discussion

The aim of this study was to investigate the relationship between regional gray matter volume and frontal cognitive impairments in the patients with ALS. Voxel‐based morphometry analysis revealed significant correlation between gray matter volume reduction in the right orbitofrontal gyrus and the total score of FAB devised to evaluate executive dysfunction. In addition, we confirmed that the physical impairments or respiratory dysfunction were not correlated with the FAB score or the orbitofrontal cortex volume. These results suggest that frontal cognitive impairments associated with the regional brain alterations occur, and the FAB is an efficient instrument to screen for frontal cognitive impairment in ALS.

The total FAB score in the ALS group was significantly lower than that in age‐matched normal controls. Of the six FAB subtests, there were significant reductions only in the similarity, lexical fluency, and go–no‐go scores. Each FAB subtest evaluates a different cognitive function under frontal lobe control, and the total FAB score gives a composite global score (Dubois et al., 2000). Therefore, in this study, the main effect of group on FAB performance was tested using the total FAB score to reveal the most salient features of global correlation patterns. In addition, frontal lobe‐mediated behavioral dysfunction was observed in apathy. These neuropsychological features in ALS were also suggested in previous reports (Ahn et al., 2011; Floris et al., 2012; Goldstein & Abrahams, 2013; Osborne et al., 2014; Raaphorst et al., 2013; Terada et al., 2011, 2016). These reports suggest that performance on executive function tests is reduced even in the relatively mild stage of ALS.

We found gray matter volume reduction in the frontal area. This pattern of brain atrophy is in accordance with previous pathologic and neuroimaging studies (Chang et al., 2005; Mezzapesa et al., 2007).

In ALS patients, reduced frontal lobe function as measured by the FAB was associated with reduced gray matter volume of the right orbitofrontal gyrus. Damage to the orbitofrontal cortex has been linked to problems with disinhibition, perseveration, impulse control, and decision making (Lovstad et al., 2012; Stout, Ready, Grace, Malloy, & Paulsen, 2003). The difficulty in controlling impulsiveness could be assessed with go–no‐go tasks, and a previous study indicated that go–no‐go scores were associated with orbital or medial frontal areas (Dubois et al., 2000; Sohn, Ursu, Anderson, Stenger, & Carter, 2000). In our study, the score on the go–no‐go subtest was associated with the gray matter volume of the right orbitofrontal gyrus. In addition, previous report indicated right dominance in go–no‐go task (Dubois et al., 2000; Garavan, Ross, & Stein, 1999; Sohn et al., 2000). This result is in line with previous studies.

The total FAB score in this study was not correlated with a change in gray matter volume in the dorsolateral or medial frontal area, which contrast with the findings of a previous report (Dubois et al., 2000), which indicated that each subtests of FAB is associated with specific areas of frontal lobe: similarity scores with dorsolateral area, lexical fluency scores with medial area (Dubois et al., 2000). We failed to show any relationship between the gray matter volume of the right orbitofrontal gyrus and similarities or lexical fluency scores. However, injury to the orbitofrontal cortex may impair aspects of verbal intelligence such as verbal fluency or performance on the similarity test by different mechanisms (e.g., perseveration). Indeed, some studies have demonstrated an association between the orbitofrontal cortex and the verbal fluency and similarity tests (Araujo et al., 2011; Lovstad et al., 2012; Ravnkilde, Videbech, Rosenberg, Gjedde, & Gade, 2002). On the other hands, the motor series score showed a significant correlation with the gray matter volume of the right orbitofrontal gyrus, whereas the motor series score was not significantly lower in ALS patients than in normal controls. Previous study indicated an association between the inferior prefrontal cortex and motor series test (Rao et al., 2008). These discrepancies might be explained by the reason that our patients had relatively mild cognitive impairment and had mild brain atrophy, and the subtests of the FAB were small range (0 to 3). Therefore, although it was thought that the association between FAB total score and the right orbitofrontal gyrus was driven by the composite characteristic of the each subtest of FAB, our findings suggest that the frontal dysfunction detected by the FAB in relatively mild‐stage ALS is mainly associated with the orbitofrontal area.

In most cases, cognitive neuroimaging studies of ALS patients have shown abnormalities mainly in the dorsolateral and medial frontal areas (Goldstein & Abrahams, 2013). However, we did not find any gray matter reductions in these areas. This might be because our ALS patients were in the relatively mild stage of disease with neither dementia nor depression.

Cognitive deficits in ALS remain challenging to evaluate because physical disability, respiratory dysfunction, or both could exaggerate performance decrements and prevent patients from completing the FAB (Ahn et al., 2011; Goldstein & Abrahams, 2013; Kim et al., 2007; Osborne et al., 2014; Raaphorst et al., 2013), given that some FAB items inquire about motor function (Dubois et al., 2000). Most previous studies did not control for physical disabilities in detail. Furthermore, significant oxygen desaturation can occur, particularly nocturnally which has shown to be related to mild cognitive change (Newsom‐Davis, Lyall, Leigh, Moxham, & Goldstein, 2001), even in individuals with seemingly adequate vital capacities (Kim et al., 2007). In an attempt to minimize the influence of physical disabilities on testing, we excluded patients with respiratory dysfunction using both respiratory function test and blood gas analytes or moderate‐to‐severe motor impairments. In addition, ALS‐related clinical factors such as the ALSFRS, respiratory test, or disease duration were not correlated with the total FAB score or the orbitofrontal cortex volume (which was positively correlated with the total FAB score). Although we did not exclude the patients with respiratory failure which starts with nocturnal hypoventilation using polysomnography, the neuroanatomical changes detected here by correlation analysis are unlikely to be explained by ALS‐related motor impairment.

Dementia and depression could also contribute to the FAB score when assessing frontal cognitive impairment (Floris et al., 2012; Goldstein & Abrahams, 2013). Therefore, we excluded subjects with obvious dementia or depression; in addition, there was no significant correlation between the right orbitofrontal gyrus volume and the MMSE or SDS score. This suggests that the neuroanatomical change related to the total FAB score would emerge in the patients with minimal depression or dementia.

However, such association between gray matter alternation and FAB score independent of physical dysfunctions could only be determined in nondemented, nondepressed ALS patients with relatively mild deficits but not in all ALS patients.

In conclusion, this study demonstrates that the frontal cognitive impairments detected by the FAB are related to gray matter atrophy in the orbitofrontal gyrus, even in patients with relatively mild‐stage ALS, and these associations are not due to motor impairment, respiratory failure, dementia, or depression. These findings also suggest that cortical ALS pathology might appear in the basal frontal lobe earlier than reported previously (Cosottini et al., 2013). Because the motor phenotype of ALS is more heterogeneous, further correlation analysis with several stages of ALS patients should be performed to determine whether any relationships between FAB score and the ALSFRS do exist. On the other hand, MMSE is heavily weighted toward memory and attention, and almost ignores executive function. Therefore, we should be cautious about describing the dementia using the MMSE. In the future, it will be necessary to investigate the neuropsychiatric symptoms in ALS patients using established neuropsychological tests. Combined neuroimaging and neuropsychological evaluation could elucidate the neural mechanisms that underlie the cognitive impairments. The FAB is easy to administer at bedside and previous report indicated that there is no significant correlation between FAB and MMSE, a measure of general cognitive function (Dubois et al., 2000). Our findings show that the FAB is a reliable tool for assessing frontal cognitive impairment in ALS. Therefore, the FAB could be helpful as a screening measure of cognitive impairment in the ALS patients. Because this study is a preliminary, cross‐sectional study, the sample size was relatively small; further researches with large samples or longitudinal studies are needed to confirm our results.

## Conflict of Interests

None.

## References

[brb3707-bib-0001] Ahn, S. W. , Kim, S. H. , Kim, J. E. , Kim, S. M. , Kim, S. H. , Sung, J. J. , … Hong, Y. H. (2011). Frontal assessment battery to evaluate frontal lobe dysfunction in ALS patients. The Canadian Journal of Neurological Sciences Le Journal Canadien des Sciences Neurologiques, 38, 242–246. 21320827 10.1017/s0317167100011409

[brb3707-bib-0002] Appollonio, I. , Leone, M. , Isella, V. , Piamarta, F. , Consoli, T. , Villa, M. L. , … Nichelli, P. (2005). The Frontal Assessment Battery (FAB): Normative values in an Italian population sample. Neurological Sciences: Official Journal of the Italian Neurological Society and of the Italian Society of Clinical Neurophysiology, 26, 108–116. 10.1007/s10072-005-0443-4 15995827

[brb3707-bib-0003] Araujo, N. B. , Barca, M. L. , Engedal, K. , Coutinho, E. S. , Deslandes, A. C. , & Laks, J. (2011). Verbal fluency in Alzheimer's disease, Parkinson's disease, and major depression. Clinics, 66, 623–627. 21655757 10.1590/S1807-59322011000400017 PMC3093793

[brb3707-bib-0004] Beghi, E. , Balzarini, C. , Bogliun, G. , Logroscino, G. , Manfredi, L. , Mazzini, L. , … Italian ALSSG (2002). Reliability of the El Escorial diagnostic criteria for amyotrophic lateral sclerosis. Neuroepidemiology, 21, 265–270. 12411728 10.1159/000065524

[brb3707-bib-0005] Cedarbaum, J. M. , Stambler, N. , Malta, E. , Fuller, C. , Hilt, D. , Thurmond, B. , & Nakanishi, A. (1999). The ALSFRS‐R: A revised ALS functional rating scale that incorporates assessments of respiratory function. BDNF ALS Study Group (Phase III). Journal of the Neurological Sciences, 169, 13–21. 10540002 10.1016/s0022-510x(99)00210-5

[brb3707-bib-0006] Chang, J. L. , Lomen‐Hoerth, C. , Murphy, J. , Henry, R. G. , Kramer, J. H. , Miller, B. L. , & Gorno‐ Tempini, M. L. (2005). A voxel‐based morphometry study of patterns of brain atrophy in ALS and ALS/FTLD. Neurology, 65, 75–80. 16009889 10.1212/01.wnl.0000167602.38643.29

[brb3707-bib-0007] Cosottini, M. , Cecchi, P. , Piazza, S. , Pesaresi, I. , Fabbri, S. , Diciotti, S. , … Bonuccelli, U. (2013). Mapping cortical degeneration in ALS with magnetization transfer ratio and voxel‐based morphometry. PLoS ONE, 8, e68279. 23874570 10.1371/journal.pone.0068279 PMC3706610

[brb3707-bib-0008] Dubois, B. , Slachevsky, A. , Litvan, I. , & Pillon, B. (2000). The FAB: A Frontal Assessment Battery at bedside. Neurology, 55, 1621–1626. 11113214 10.1212/wnl.55.11.1621

[brb3707-bib-0009] Floris, G. , Borghero, G. , Chio, A. , Secchi, L. , Cannas, A. , Sardu, C. , … Marrosu, M. G. (2012). Cognitive screening in patients with amyotrophic lateral sclerosis in early stages. Amyotrophic Lateral Sclerosis: Official Publication of the World Federation of Neurology Research Group on Motor Neuron Diseases, 13, 95–101. 10.3109/17482968.2011.605453 21895509

[brb3707-bib-0010] Garavan, H. , Ross, T. J. , & Stein, E. A. (1999). Right hemispheric dominance of inhibitory control: An event‐related functional MRI study. Proceedings of the National Academy of Sciences of the United States of America, 96, 8301–8306. 10393989 10.1073/pnas.96.14.8301 PMC22229

[brb3707-bib-0011] Goldstein, L. H. , & Abrahams, S. (2013). Changes in cognition and behaviour in amyotrophic lateral sclerosis: Nature of impairment and implications for assessment. The Lancet Neurology, 12, 368–380. 23518330 10.1016/S1474-4422(13)70026-7

[brb3707-bib-0012] Grace, J. , Stout, J. C. , & Malloy, P. F. (1999). Assessing frontal lobe behavioral syndromes with the frontal lobe personality scale. Assessment, 6, 269–284. 10445964 10.1177/107319119900600307

[brb3707-bib-0013] Holmes, C. B. , Fouty, H. E. , Wurtz, P. J. , & Burdick, B. M. (1988). Zung Self‐Rating Depression Scale scores of psychiatric outpatients by age and sex. Psychological Reports, 62, 259–262. 3363063 10.2466/pr0.1988.62.1.259

[brb3707-bib-0014] Hughes, C. P. , Berg, L. , Danziger, W. L. , Coben, L. A. , & Martin, R. L. (1982). A new clinical scale for the staging of dementia. The British Journal of Psychiatry: The Journal of Mental Science, 140, 566–572. 7104545 10.1192/bjp.140.6.566

[brb3707-bib-0015] Kim, S. M. , Lee, K. M. , Hong, Y. H. , Park, K. S. , Yang, J. H. , Nam, H. W. , … Lee, K. W. (2007). Relation between cognitive dysfunction and reduced vital capacity in amyotrophic lateral sclerosis. Journal of Neurology, Neurosurgery, and Psychiatry, 78, 1387–1389. 10.1136/jnnp.2006.111195 PMC2095584 17557798

[brb3707-bib-0016] Kubota, M. , Miyata, J. , Hirao, K. , Fujiwara, H. , Kawada, R. , Fujimoto, S. , … Murai, T. (2011). Alexithymia and regional gray matter alterations in schizophrenia. Neuroscience Research, 70, 206–213. 21300113 10.1016/j.neures.2011.01.019

[brb3707-bib-0017] Kugo, A. , Terada, S. , Ata, T. , Ido, Y. , Kado, Y. , Ishihara, T. , … Kuroda, S. (2007). Japanese version of the Frontal Assessment Battery for dementia. Psychiatry Research, 153, 69–75. 17599465 10.1016/j.psychres.2006.04.004

[brb3707-bib-0018] Kume, K. , Hanyu, H. , Murakami, M. , Sato, T. , Hirao, K. , Kanetaka, H. , … Iwamoto, T. (2011). Frontal Assessment Battery and brain perfusion images in amnestic mild cognitive impairment. Geriatrics and Gerontology International, 11, 77–82. 20807243 10.1111/j.1447-0594.2010.00645.x

[brb3707-bib-0019] Lomen‐Hoerth, C. , Murphy, J. , Langmore, S. , Kramer, J. H. , Olney, R. K. , & Miller, B. (2003). Are amyotrophic lateral sclerosis patients cognitively normal? Neurology, 60, 1094–1097. 12682312 10.1212/01.wnl.0000055861.95202.8d

[brb3707-bib-0020] Lovstad, M. , Funderud, I. , Endestad, T. , Due‐Tonnessen, P. , Meling, T. R. , Lindgren, M. , … Solbakk, A. K. (2012). Executive functions after orbital or lateral prefrontal lesions: Neuropsychological profiles and self‐reported executive functions in everyday living. Brain Injury, 26, 1586–1598. 22731818 10.3109/02699052.2012.698787 PMC4090100

[brb3707-bib-0021] Matsui, H. , Udaka, F. , Miyoshi, T. , Hara, N. , Tamura, A. , Oda, M. , … Kameyama, M. (2006). Frontal assessment battery and brain perfusion image in Parkinson's disease. Journal of Geriatric Psychiatry and Neurology, 19, 41–45. 16449760 10.1177/0891988705284714

[brb3707-bib-0022] Mezzapesa, D. M. , Ceccarelli, A. , Dicuonzo, F. , Carella, A. , De Caro, M. F. , Lopez, M. , … Simone, I. L. (2007). Whole‐brain and regional brain atrophy in amyotrophic lateral sclerosis. AJNR American Journal of Neuroradiology, 28, 255–259. 17296989 PMC7977419

[brb3707-bib-0023] Miyata, J. , Hirao, K. , Namiki, C. , Fujiwara, H. , Shimizu, M. , Fukuyama, H. , … Murai, T. (2009). Reduced white matter integrity correlated with cortico‐subcortical gray matter deficits in schizophrenia. Schizophrenia Research, 111, 78–85. 19361957 10.1016/j.schres.2009.03.010

[brb3707-bib-0024] Murphy, J. , Henry, R. , & Lomen‐Hoerth, C. (2007). Establishing subtypes of the continuum of frontal lobe impairment in amyotrophic lateral sclerosis. Archives of Neurology, 64, 330–334. 17353375 10.1001/archneur.64.3.330

[brb3707-bib-0025] Nakaaki, S. , Murata, Y. , Sato, J. , Shinagawa, Y. , Matsui, T. , Tatsumi, H. , & Furukawa, T. A. (2007). Reliability and validity of the Japanese version of the Frontal Assessment Battery in patients with the frontal variant of frontotemporal dementia. Psychiatry and Clinical Neurosciences, 61, 78–83. 17239043 10.1111/j.1440-1819.2007.01614.x

[brb3707-bib-0026] Neary, D. , Snowden, J. S. , Gustafson, L. , Passant, U. , Stuss, D. , Black, S. , … Benson, D. F. (1998). Frontotemporal lobar degeneration: A consensus on clinical diagnostic criteria. Neurology, 51, 1546–1554. 9855500 10.1212/wnl.51.6.1546

[brb3707-bib-0027] Newsom‐Davis, I. C. , Lyall, R. A. , Leigh, P. N. , Moxham, J. , & Goldstein, L. H. (2001). The effect of non‐invasive positive pressure ventilation (NIPPV) on cognitive function in amyotrophic lateral sclerosis (ALS): A prospective study. Journal of Neurology, Neurosurgery, and Psychiatry, 71, 482–487. 10.1136/jnnp.71.4.482 PMC1763518 11561031

[brb3707-bib-0028] Osborne, R. A. , Sekhon, R. , Johnston, W. , & Kalra, S. (2014). Screening for frontal lobe and general cognitive impairment in patients with amyotrophic lateral sclerosis. Journal of the Neurological Sciences, 336, 191–196. 24239183 10.1016/j.jns.2013.10.038

[brb3707-bib-0029] Raaphorst, J. , Beeldman, E. , Jaeger, B. , Schmand, B. , van den Berg, L. H. , Weikamp, J. G. , … de Haan, R. J. (2013). Is the Frontal Assessment Battery reliable in ALS patients? Amyotrophic Lateral Sclerosis and Frontotemporal Degeneration, 14, 73–74. 22889176 10.3109/17482968.2012.712974

[brb3707-bib-0030] Rao, H. , Di, X. , Chan, R. C. , Ding, Y. , Ye, B. , & Gao, D. (2008). A regulation role of the prefrontal cortex in the fist‐edge‐palm task: Evidence from functional connectivity analysis. NeuroImage, 41, 1345–1351. 18495496 10.1016/j.neuroimage.2008.04.026

[brb3707-bib-0031] Ravnkilde, B. , Videbech, P. , Rosenberg, R. , Gjedde, A. , & Gade, A. (2002). Putative tests of frontal lobe function: A PET‐study of brain activation during Stroop's Test and verbal fluency. Journal of Clinical and Experimental Neuropsychology, 24, 534–547. 12187466 10.1076/jcen.24.4.534.1033

[brb3707-bib-0032] Sohn, M. H. , Ursu, S. , Anderson, J. R. , Stenger, V. A. , & Carter, C. S. (2000). The role of prefrontal cortex and posterior parietal cortex in task switching. Proceedings of the National Academy of Sciences of the United States of America, 97, 13448–13453. 11069306 10.1073/pnas.240460497 PMC27244

[brb3707-bib-0033] Stout, J. C. , Ready, R. E. , Grace, J. , Malloy, P. F. , & Paulsen, J. S. (2003). Factor analysis of the frontal systems behavior scale (FrSBe). Assessment, 10, 79–85. 12675387 10.1177/1073191102250339

[brb3707-bib-0034] Talairach, J. , & Tournoux, P. (1988). Co‐planar stereotaxic atlas of the human brain: 3‐dimensional proportional system: An approach to cerebral imaging, 122 pp. Stuttgart, New York, NY: Georg Thieme.

[brb3707-bib-0035] Terada, T. , Obi, T. , Miyata, J. , Kubota, M. , Yoshizui, M. , Murai, T. , … Mizoguchi, K. (2016). Correlation of frontal atrophy with behavioral changes in amyotrophic lateral sclerosis. Neurology and Clinical Neuroscience, 4, 85–92.

[brb3707-bib-0036] Terada, T. , Obi, T. , Yoshizumi, M. , Murai, T. , Miyajima, H. , & Mizoguchi, K. (2011). Frontal lobe‐mediated behavioral changes in amyotrophic lateral sclerosis: Are they independent of physical disabilities? Journal of the Neurological Sciences, 309, 136–140. 21782199 10.1016/j.jns.2011.06.049

[brb3707-bib-0037] Tombaugh, T. N. , & McIntyre, N. J. (1992). The mini‐mental state examination: A comprehensive review. Journal of the American Geriatrics Society, 40, 922–935. 1512391 10.1111/j.1532-5415.1992.tb01992.x

[brb3707-bib-0038] Ueda, K. , Fujiwara, H. , Miyata, J. , Hirao, K. , Saze, T. , Kawada, R. , … Murai, T. (2010). Investigating association of brain volumes with intracranial capacity in schizophrenia. NeuroImage, 49, 2503–2508. 19770046 10.1016/j.neuroimage.2009.09.006

[brb3707-bib-0039] Yoshizumi, M. , Ueda, K. , Ohigashi, Y. , & Murai, T. (2007). Reliability, validity, and standardization of the Japanese version of the Frontal Systems Behavioral Scale. Seishin Igaku, 49, 137–142.

